# The Volume and Tone of Twitter Posts About Cannabis Use During Pregnancy: Protocol for a Scoping Review

**DOI:** 10.2196/34421

**Published:** 2022-03-29

**Authors:** Liam Cresswell, Lisette Espin-Noboa, Malia S Q Murphy, Serine Ramlawi, Mark C Walker, Márton Karsai, Daniel J Corsi

**Affiliations:** 1 Obstetrics & Maternal Newborn Investigations Research Group Clinical Epidemiology Program Ottawa Hospital Research Institute Ottawa, ON Canada; 2 Faculty of Medicine University of Ottawa Ottawa, ON Canada; 3 Department of Network and Data Science Central European University Vienna Austria; 4 Department of Obstetrics and Gynecology Faculty of Medicine University of Ottawa Ottawa, ON Canada; 5 School of Epidemiology and Public Health University of Ottawa Ottawa, ON Canada; 6 Better Outcomes Registry & Network Ontario Ottawa, ON Canada; 7 International and Global Health Office University of Ottawa Ottawa, ON Canada; 8 CHEO Research Institute Ottawa, ON Canada

**Keywords:** cannabis, pregnancy, health information, social media, Twitter

## Abstract

**Background:**

Cannabis use has increased in Canada since its legalization in 2018, including among pregnant women who may be motivated to use cannabis to reduce symptoms of nausea and vomiting. However, a growing body of research suggests that cannabis use during pregnancy may harm the developing fetus. As a result, patients increasingly seek medical advice from online sources, but these platforms may also spread anecdotal descriptions or misinformation. Given the possible disconnect between online messaging and evidence-based research about the effects of cannabis use during pregnancy, there is a potential for advice taken from social media to affect the health of mothers and their babies.

**Objective:**

This study aims to quantify the volume and tone of English language posts related to cannabis use in pregnancy from January 2012 to December 2021.

**Methods:**

Modeling published frameworks for scoping reviews, we will collect publicly available posts from Twitter that mention cannabis use during pregnancy and use the Twitter Application Programming Interface for Academic Research to extract data from tweets, including public metrics such as the number of likes, retweets, and quotes, as well as health effect mentions, sentiment, location, and users’ interests. These data will be used to quantify how cannabis use during pregnancy is discussed on Twitter and to build a qualitative profile of supportive and opposing posters.

**Results:**

The CHEO Research Ethics Board reviewed our project and granted an exemption in May 2021. As of December 2021, we have gained approval to use the Twitter Application Programming Interface for Academic Research and have developed a preliminary search strategy that returns over 3 million unique tweets posted between 2012 and 2021.

**Conclusions:**

Understanding how Twitter is being used to discuss cannabis use during pregnancy will help public health agencies and health care providers assess the messaging patients may be receiving and develop communication strategies to counter misinformation, especially in geographical regions where legalization is recent or imminent. Most importantly, we foresee that our findings will assist expecting families in making informed choices about where they choose to access advice about using cannabis during pregnancy.

**Trial Registration:**

Open Science Framework 10.17605/OSF.IO/BW8DA; www.osf.io/6fb2e

**International Registered Report Identifier (IRRID):**

PRR1-10.2196/34421

## Introduction

Recreational cannabis use has increased in Canada since its legalization in 2018, including among pregnant women [1]. Reductions in the perceived harms of cannabis use may occur around legalization, and as a result, pregnant women or individuals may find the activity to be low risk [2]. Cannabis and its derivative products are often marketed online as safe [3]. Certain groups and dispensaries may even promote the use of cannabis products during pregnancy for their antinausea and antiemetic effects [4,5]. Expecting mothers may also use the drug to stimulate appetite or treat depression, motivated by the perception that cannabis is natural and thus preferable to prescription medications [6]. However, a growing body of research suggests that cannabis and derivative products during pregnancy may harm the developing fetus. Cannabinoids readily cross the placenta and interfere with the endogenous cannabinoid system, a cell-signaling network that assists in neurodevelopment [7]. Consequently, maternal cannabis use has been associated with fetal growth restriction, higher rates of childhood affective disorders, and a greater incidence of learning disability and autism spectrum disorders among offspring [8-10].

Pregnant patients increasingly seek medical and health advice on online platforms, especially for emerging topics like cannabis use [11,12]. Although medical professionals and research groups may use these avenues to promote research findings, other Twitter users may use social media to promote commercial interests, share anecdotal stories, or spread misinformation [13-15]. For example, a 2019 study by Ishida et al [16] found that those who primarily rely on social media for their health information were 31% more likely than others to endorse the claim that cannabis use during pregnancy is safe and 56% more likely to endorse any form of misinformation about cannabis.

Given the possible disconnect between online messaging and evidence-based research about the effects of cannabis use during pregnancy, there is the possibility that advice taken from social media could have inaccuracies that may affect the health of mothers and their babies. Here, we propose a systematic search of Twitter to quantify the volume and tone of posts on the forum related to cannabis use in pregnancy. Twitter is a global platform, and our findings may have relevance in Canada, the United States, and other jurisdictions where access and availability to cannabis are increasing due to legalization. We will assess regional correlations in these data to determine if changes in the legalization of nonmedical cannabis affect online messaging of its use during pregnancy in Canada and states in the United States that have legalized recreational cannabis.

## Methods

### Overview

With reference to Arksey and O’Malley’s [17] framework for scoping reviews, we will synthesize publicly available posts from Twitter to determine how cannabis use during pregnancy is being discussed on the platform [17]. The steps, as outlined by this framework and adapted for a Twitter-based analysis, will be:

- Identifying the research question
- Identifying relevant Twitter posts
- Selecting eligible Twitter posts
- Charting the data
- Collating, summarizing, and reporting the results

Past research from Cavazos-Rehg et al [18] has identified Twitter as a good source for analyzing online discussions about cannabis use because of its popularity and acceptance of substance use disclosure. We will use this to model a novel scoping review approach to explore Twitter posts about cannabis use during pregnancy. We will report our findings following the PRISMA-ScR (Preferred Reporting Items for Systematic Reviews and Meta-Analyses Extension for Scoping Reviews) [19].

### Step 1: Identifying the Research Question

How is cannabis use during pregnancy discussed on Twitter regarding the volume, tone, content, and authors/users?

### Step 2: Identifying Relevant Twitter Posts

Our search strategy will follow an iterative approach according to our population, concept, and context of interest (Textbox 1). We will first use Twitter’s native search function to conduct a preliminary scan of English language tweets about cannabis use during pregnancy and assemble a list of commonly used keywords and hashtags based on our findings. We will then refine our list to capture the breadth of online discussion while excluding mimicker terms (eg, non–drug-related uses of the word “high”). Finally, our search strategy will include a list of terms for pregnancy combined with terms for cannabis to search the Twitter Application Programming Interface (API; Textbox 2), for example, (pregnancy OR pregnant OR prenatal) AND (cannabis OR weed OR pot OR marijuana), with the final search strategy to be developed following preliminary findings. We will use the Twitter API for Academic Research for data collection. We will perform a full archive search of all English language tweets containing the keywords of interest posted from January 2012, when Colorado became the first English-speaking jurisdiction to legalize cannabis, to December 2021 [20].

**Population, Concept, Context framework.**

**Population**

Twitter posts containing information relevant to pregnancy or pregnant individuals

**Concept**

Discussion or mention of cannabis use in relation to pregnancy or the developing fetus

**Context**

All English language Twitter posts (tweets) made from January 2012 to December 2021. Geographical analyses will be restricted to Canada and states in the United States where recreational cannabis use is legal.

**List of keywords related to cannabis use in pregnancy used to search the Twitter Application Programming Interface.**

**Pregnancy related **

Pregnancy, pregnant, baby, fetus, fetal, prenatal, perinatal, womb, preggo, “pregnant life,” “baby bump,” “mom to be,” “mommy to be,” “baby on the way,” “preggers,” “pregnant af”

**Cannabis related**

cannabis, weed, pot, marijuana, marihuana, MJ, ganja, purp, bud, keef, kief, dope, “mary jane,” thc, cbd, cannamom, opiate, mdma, ecstasy, mmj, medical marijuana, blunt, bong, budder, hash, hemp, indica, kush, reefer, sativa

### Step 3: Selecting Eligible Twitter Posts

**Figure 1:**
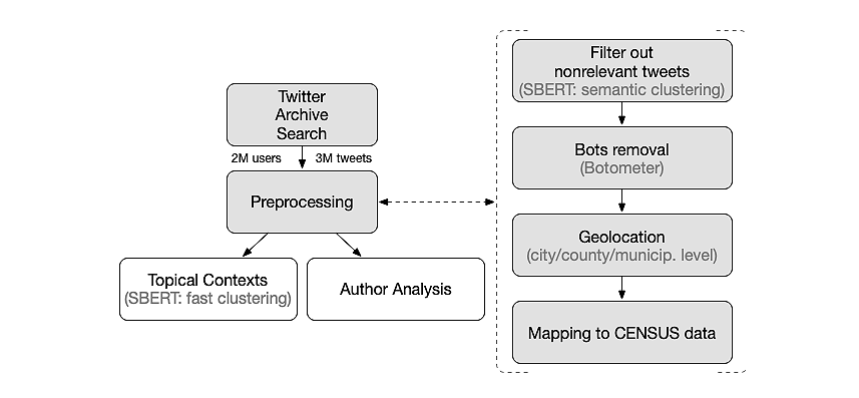
Overview of the proposed data collection methodology, preprocessing, and analytical process for tweets about cannabis use during pregnancy. SBERT: Sentence Bidirectional Encoder Representations from Transformers.

Following the Twitter Archive search, we will preprocess the corpora to filter out content unrelated to *cannabis use during pregnancy*. Additionally, we will remove bot accounts [21], and tweets without geotags will be further analyzed to infer a location from their authors’ profile [22,23] (Figure 1). We will filter out all tweets containing our keywords but that are unrelated to the consumption of cannabis during pregnancy via a *symmetric semantic search* using Sentence Bidirectional Encoder Representations from Transformers (BERT) [24]. This search assigns a score to each tweet for each given query (Textbox 3). The higher the score, the more semantically close the tweet is to the query. Tweets with a score lower than, for example, 0.6 for all queries are discarded since they are likely unrelated. The cutoff value of 0.6 was selected here for illustrative purposes. In the final analysis, we will tune this parameter and select the score that gives optimal classification results. We will also perform a topical context analysis to provide meaning and classify tweets by performing a *semantic community detection* using Sentence BERT [24]. We will use the “Fast clustering” algorithm together with “all-MiniLM-L6-v2” a pretrained sentence-transformer model for large-scale data sets [25]. In this model, we will set the minimum size of communities (or clusters) to 10 and a threshold similarity of 0.6. In other words, clusters will contain at least 10 tweets, and the similarity between tweets of the same cluster will be at least 60%. We will further classify related tweets into broad categories related to cannabis during pregnancy and medical cannabis or cannabis and youth, or legalization of cannabis. In addition, we will classify tweets related to cannabis during pregnancy into commercial, anecdotal/conversational/babble, misinformation, memes, and research studies.

Note, that most irrelevant tweets are pruned out by Sentence BERT in the preprocessing phase (Figure 1). We will evaluate the accuracy of this filtering by randomly sampling both types of tweets and label them as relevant or irrelevant by three independent reviewers and report precision and recall based on majority voting. Similarly, in the clustering phase, we will revise the inferred clusters and merge (if necessary) those that might be related to the same topical context.

**Queries passed to the symmetric semantic search of Sentence Bidirectional Encoder Representations from Transformers.**

**Queries**

- Cannabis during pregnancy
- Kids, children, and youth smoking cannabis
- Smoking cannabis while pregnant
- Medical cannabis for people
- The effects of cannabis on pregnant women
- Legalization of cannabis
- Smoking or consuming drugs during pregnancy

### Step 4: Charting the Data

Data charting will include an automated analysis of all tweets returned by our search. A manual analysis will then be conducted on the smaller subset of tweets included during the process outlined in Step 2.

Using the Twitter API for Academic Research [26], we will collect the timestamp of each returned tweet and analyze its text for sentiment (positive or negative) by integrating with the Natural Language Toolkit in Python and other techniques such as latent Dirichlet allocation [27], Sentence BERT [24], or recurrent neural networks [28]. We will also analyze the number and types of health effects mentioned in association with cannabis use in pregnancy and will extract location data when available from each tweet, either from geotagged tweets or from the location associated with the user’s profile [29].

Three independent reviewers will manually review the smaller subset of randomly sampled tweets. We will verify the number of favorites and retweets each tweet has received against the automatic data collection via the API. We will use publicly available user lists to determine the category of organization or individual user that posted the tweet (government or public health agency, obstetrical society/network, university, hospital, news outlet, cannabis industry source, or other individual) [30], and we will manually (via majority voting from three reviewers) assign a category for organizations not appearing on the user lists. Finally, we will assess if the tweet mentions positive or adverse health effects on mothers or developing fetus/infants, and the specific health effects mentioned. For each tweet, data will be extracted by one reviewer and validated by a second reviewer. A third independent reviewer will resolve discrepancies if they arise.

Separately, we will also extract CENSUS or population-level data on birth rates and maternal and infant mortality rates across the study period in Canada and the United States. It has been shown that Twitter is a good proxy to infer health-related statistics, including teenage birth rates [31]. Thus, we want to verify whether certain geographical areas with certain CENSUS characteristics behave similarly with respect to their opinions on cannabis use during pregnancy. These vital statistics data will be sourced from Statistics Canada and the Centers for Disease Control and Prevention in the United States [32,33].

### Step 5: Collating, Summarizing, and Reporting the Results

We will first report the total number of tweets returned over the search period and temporal trends in the number of tweets posted over the study period. Next, the number of tweets sampled in the automated and manual analyses will be reported. From the automated analysis, we will report the number and percentage of the returned posts that discuss cannabis use during pregnancy positively or negatively as determined by our sentiment analysis. Subsequently, we will calculate the standardized mean difference in the number of favorites and retweets received by positive and negative tweets, and to compute the odds (ratio) that positive posts originate from each category of organization or individual and mention health effects. We will further calculate the number of times each health effect was mentioned as a percentage of the total health effect mentions. These statistics will be presented in tabular form.

The location-based component of our analysis will be restricted to tweets that offer location data and originate from Canada and legal states within the United States, as these are the only English-speaking regions that have legalized the sale of nonmedical cannabis. If any regions (eg, New Zealand or the United Kingdom) legalize cannabis before our analysis is conducted, this restriction will be changed to include them. We will match location data from these jurisdictions to the timestamp for each tweet to calculate the proportion of tweets originating from our predefined geographical regions for each week of the search period. Next, we will visualize each region on a line graph that plots time versus the volume of posts with a marker to indicate when that region legalized cannabis. A line graph that plots time versus percentage of positive posts will be plotted using the same process. We will then use a repeated cross-sectional design to analyze the correlation of these data with population-level vital statistics data and determine if trends in cannabis messaging on Twitter correlate with birth rates and maternal and infant mortality rates.

In addition to these numerical analyses, we will develop qualitative profiles of influential accounts. These profiles will include elements such as the user’s background (eg, political leaning, socioeconomic status, or education/interests); their Twitter following; whether Twitter has verified their account as “authentic, notable, and active” [34]; and how they contribute to the discussion about cannabis use during pregnancy on the platform. Comparisons and contrasts will be drawn between the typical supportive and opposing posters based on these elements.

### Ethics and Dissemination

This study was exempted from ethics review on the basis that it will collect and synthesize publicly available data. Therefore, the research does not require ethical approval.

## Results

**Figure 2:**
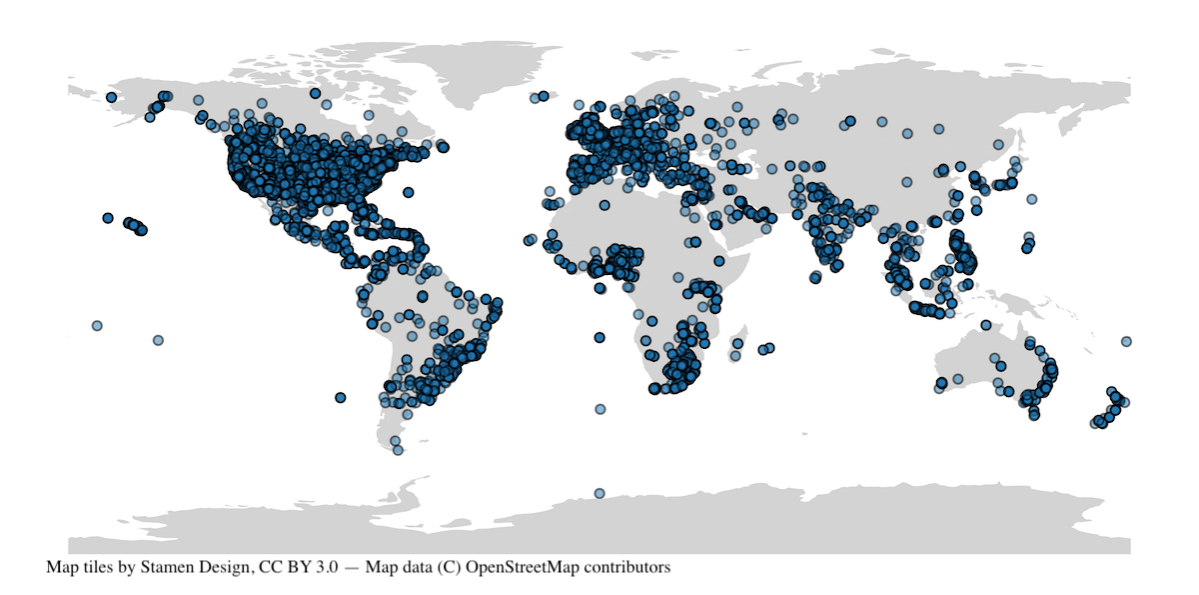
Geographic distribution of geotagged tweets containing pregnancy and cannabis-related keywords posted between January 1, 2012, to December 31, 2021.

**Figure 3:**
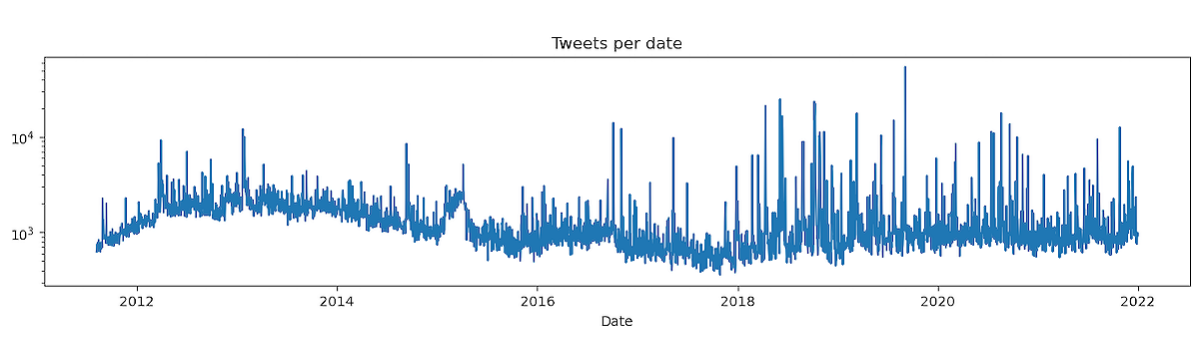
Number of tweets per day related to cannabis in pregnancy, January 1, 2012, to December 31, 2021.

Using our data collection method, combing the search_all_tweets function from Tweepy [35] together with the Twitter API for Academic Research, we collected 2,000,000 tweets and 1,000,000 retweets that are potentially related to cannabis use during pregnancy. These results cover all English language tweets posted from January 1, 2012, to December 31, 2021 (10 years), that include both *pregnancy*- and *cannabis*-related keywords. Of the 3,000,000 unique tweets, only 4.3% of them are geotagged (Figure 2). Note that these tweets are concentrated mainly in English-speaking cities or countries. This finding is expected since our search explicitly requested English tweets. Figure 3 shows the frequency distribution of all 3,000,000 tweets per day since 2012. Colorado was the first English-speaking jurisdiction to legalize cannabis in 2012, and Canada legalized cannabis in 2018. Our Twitter search includes 47 distinct keywords; we plotted the number of times each keyword appears in our corpora (Table 1).

**Table 1:** Frequency of cannabis-related keywords identified in tweets posted between January 1, 2012, to December 31, 2021.

Keyword	Count
weed	1,047,115
dope	688,153
blunt	556,865
pot	399,444
keef	356,605
marijuana	183,409
bud	161,328
bong	116,876
kush	99,916
thc	44,970
hash	44,906
cbd	39,287
ecstasy	33,989
hemp	28,514
purp	25,353
ganja	24,641
indica	8447
reefer	6125
opiate	4102
kief	3092
mdma	2459
mmj	1386
budder	643
marihuana	637
cannamom	40
medicalmarijuana	13

**Figure 4:**
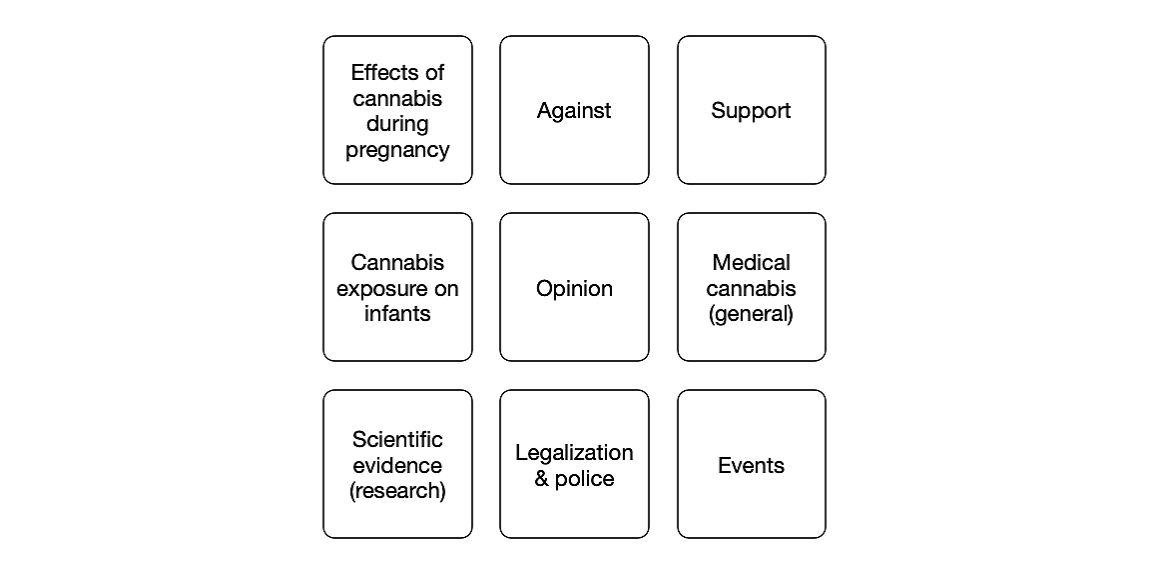
Topical contexts (clusters) identified from tweets collected about cannabis use during pregnancy.

The semantic community detection algorithm detected 220 clusters within the 3,000,000 tweets from our corpora. We manually inspected the top 5 and bottom 5 tweets of each cluster and assigned an appropriate label that best described the topical context of those tweets. For example, we found 9 topical clusters related to *cannabis use during pregnancy* (Figure 4). A sample of paraphrased tweets from one identified cluster, “Cannabis exposure on infants,” is shown (Table 2).

We expect to conclude this study in December 2022.

**Table 2:** Top 3 and bottom 3 tweets selected from the cluster “Cannabis exposure on infants.”^a^

No.	Paraphrased tweet
1	random *thc* found in *baby* soap
2	newborns test positive *marijuana* from *baby* soap
3	*marijuana* in newborns from *baby* soap are false positives.
45	*Baby* you only do *thc*, you need help.
46	pediatric doctor advises passing *thc* via placenta and breast feeding (previously thought to damage *baby* brain)
47	expert on *thc* exposure during pregnancy is Dr. X at Clinic Y.

^a^Italicized words represent our set of query keywords.

## Discussion

### Preliminary Findings

This study will infer how cannabis use in pregnancy is portrayed on Twitter, the content and origin of supportive posts, and how legal status changes influence the volume and tone of posts related to cannabis in pregnancy. Our findings will help inform policy strategies to public health agencies, care providers, and other stakeholders. Moreover, they will suggest future avenues for research. Our preliminary findings suggest that this work is feasible and that we have identified a sufficiently robust corpus of tweets for more detailed analyses.

### Limitations and Future Work

Twitter is an extensive online platform to share news and opinions [36]. However, it is not representative of the whole population [37]. A 2016 survey found that only 21% of Americans use Twitter [38]. Users are, on average, younger and better educated than nonusers, and they are more liberal and pay more attention to politics [37]. However, a recent study [39] has shown that young adults (25-44 years) that were active on an abortion debate on Twitter were well represented compared to the 2017 CENSUS representation in Chile. While this age range overlaps with the women’s reproductive age (15-44 years), birth rates decreased for females aged 15 to 34 years, increased for females aged 35 to 44 years, and were unchanged for females aged 10 to 14 years and 45 to 49 years from 2018 to 2019 in the United States [40].

Besides Twitter, there are several online platforms used to share opinions, for instance, Facebook, Reddit, and Quora. To the best of our knowledge, only Facebook has been used to study people’s opinions on cannabis [41] and during pregnancy [42]. However, in these studies, authors run surveys by targeting people via Facebook ads (ie, findings are based on answers to questionaries) and did not analyze free-text opinions. Here, we opt to use Twitter data since it has been shown that there is rich content to study health-related issues [20,43], including opinions on the use of cannabis during pregnancy [44-46]. Besides, Twitter is one of the largest social media platforms allowing discussions and debates with 187,000,000 daily users [47]. Future research may focus on other platforms to study how people discuss cannabis use during pregnancy and verify whether all these users combined can make a better representation of their offline population.

### Conclusions

We will submit the final results of our review for publication in a peer-reviewed journal, present at academic conferences, and share through publicly available streams such as the professional and institutional social media accounts and webpages associated with the research team. The results will provide insight into how frequently and in what context Twitter is being used to discuss cannabis use during pregnancy. We anticipate that this knowledge will help public health agencies and health care providers assess the messaging patients may be receiving on Twitter and develop communication strategies to counter misinformation, especially in geographical regions where legalization is recent or imminent. Most importantly, we foresee that our findings will assist expecting families in making informed choices about where they choose to access advice about using cannabis during pregnancy.

## Abbreviations

API: Application Programming Interface
BERT: Bidirectional Encoder Representations from Transformers
PRISMA-ScR: Preferred Reporting Items for Systematic Reviews and Meta-Analyses Extension for Scoping Reviews
